# Rice NAC transcription factor ONAC095 plays opposite roles in drought and cold stress tolerance

**DOI:** 10.1186/s12870-016-0897-y

**Published:** 2016-09-20

**Authors:** Lei Huang, Yongbo Hong, Huijuan Zhang, Dayong Li, Fengming Song

**Affiliations:** National Key Laboratory for Rice Biology, Institute of Biotechnology, Zhejiang University, Hangzhou, 310058 People’s Republic of China

**Keywords:** Abscisic acid (ABA), Cold tolerance, Drought tolerance, NAC transcription factor, ONAC095, Rice (*Oryza sativa* L.)

## Abstract

**Background:**

The NAC (NAM, ATAF and CUC) transcriptional factors constitute a large family with more than 150 members in rice and some of them have been demonstrated to play crucial roles in plant abiotic stress response. Here, we report the characterization of a rice stress-responsive NAC gene, *ONAC095*, and the exploration of its function in drought and cold stress tolerance.

**Results:**

Expression of *ONAC095* was up-regulated by drought stress and abscisic acid (ABA) but down-regulated by cold stress. ONAC095 protein had transactivation activity and the C2 domain in C-terminal was found to be critical for transactivation activity. Transgenic rice lines with overexpression of *ONAC095* (ONAC095-OE) and dominant chimeric repressor-mediated suppression of *ONAC095* (ONAC095-SRDX) were generated. The ONAC095-OE plants showed comparable phenotype to wild type under drought and cold stress conditions. However, the ONAC095-SRDX plants displayed an improved drought tolerance but exhibited an attenuated cold tolerance. The ONAC095-SRDX plants had decreased water loss rate, increased proline and soluble sugar contents, and up-regulated expression of drought-responsive genes under drought condition, whereas the ONAC095-SRDX plants accumulated excess reactive oxygen species, increased malondialdehyde content and down-regulated expression of cold-responsive genes under cold condition. Furthermore, ONAC095-SRDX plants showed an increased ABA sensitivity, contained an elevated ABA level, and displayed altered expression of ABA biosynthetic and metabolic genes as well as some ABA signaling-related genes.

**Conclusion:**

Functional analyses through dominant chimeric repressor-mediated suppression of *ONAC095* demonstrate that ONAC095 plays opposite roles in drought and cold stress tolerance, acting as a negative regulator of drought response but as a positive regulator of cold response in rice.

**Electronic supplementary material:**

The online version of this article (doi:10.1186/s12870-016-0897-y) contains supplementary material, which is available to authorized users.

## Background

Environmental constraints such as drought, salt and extreme temperatures often affect adversely plant growth and development, which lead to great loss of productivity worldwide [1]. Extensive studies have revealed that plants can timely sense external signals and initiate effectively complicated signaling networks to respond to environmental stress by activating various cellular, physiological, biochemical and metabolic processes [2–4]. Abscisic acid (ABA), as a critical stress phytohormone, plays important roles in abiotic stress signaling networks, and the ABA-mediated stress signaling can be divided into ABA-dependent and ABA-independent pathways [4, 5]. A number of key genes that are involved in the ABA-dependent and ABA-independent stress pathways have been identified, including DRE-binding protein/C-repeat-binding factor (CBF), ABA-binding factor, MYC and MYB [4, 5]. In addition, stress-induced reactive oxygen species (ROS), including hydrogen peroxide (H_2_O_2_) and superoxide anion, are harmful by-products causing cellular oxidative damage at excess level [6], whereas ROS are also considered to play signaling roles in regulating abiotic stress response at proper cellular concentration [6–8].

Upon perception of environmental stimuli, stress-initiated signaling network often effectively and timely reprograms the expression of a large spectrum of stress-responsive genes [4, 9, 10]. For example, a total of 5866 genes (2145 up-regulated and 3721 down-regulated), accounting for ~18 % of the annotated genes in rice genome, were differentially expressed during drought stress in rice [11]. Such large proportion of differentially expressed genes during a specific abiotic stress response requires a synergistic action of different types of transcription factors (TFs) in both temporal and spatial manners. Genetic and molecular studies using knockout/knockdown mutants and/or overexpression lines have revealed that many families of TFs such as NAC, AP2/ERF, MYB, WRKY, bZIP, homeodomain, bHLH, NF-Y and CAMTA have members that play roles in abiotic stress response [12–17]. It was also suggested that some of the functionally characterized TF genes may have great potentials in improvement of abiotic stress tolerance in crop plants [18].

NAC proteins are plant-specific TFs [19] and constitute a large family with 151 members in rice [20–22]. The NAC TFs contain a highly conserved NAC domain at N-terminal, which determines DNA-binding activity, and a variable domain at C-terminal, which is responsible for transcription activity [19]. Beside the involvement in growth and development [23], the function of the NAC TFs in biotic and abiotic stress responses has been well documented in both model and crop plants [14, 15, 17, 24]. Transcriptional profiling revealed that a relative large portion of the Arabidopsis and rice NAC TF families exhibited differential expression patterns in response to various biotic and abiotic stresses [25–27]. Up to date, six rice NAC genes, e.g. *ONAC048* (*OsNAC6*), *ONAC048* (*OsNAC111*), *ONAC122*, *ONAC131*, *ONAC054* (*RIM1*) and *ONAC068* (*OsNAC4*), have been reported to be involved in pathogen defense response [28–31]. Meanwhile, at least 7 NAC genes including *ONAC002* (*SANC1*/*OsNAC9*), *ONAC048* (*SNAC2*/*OsNAC6*), *ONAC009* (*OsNAC5*), *ONAC122* (*OsNAC10*), *ONAC045*, *ONAC058* (*OsNAP*) and *ONAC022* have been shown to play roles in abiotic stress tolerance [32–42]. Overexpression of some of these NAC TF genes in transgenic rice improved significantly the drought and salinity tolerance and the ABA-mediated signaling pathway [32, 33, 35, 39–41], stomatal movement and root system [32, 35, 37–39] are involved in the improved abiotic stress tolerance in the transgenic plants.

In our previous study, a number of stress-responsive *ONAC* genes in rice response to biotic and abiotic stresses were identified through analysis of publicly available microarray data [26, 28]. In the present study, we performed a detailed functional analysis of *ONAC095* in abiotic stress tolerance by overexpression and dominant chimeric repressor-mediated suppression of *ONAC095* in transgenic rice. Our results revealed that dominant chimeric repressor-mediated suppression of *ONAC095* function confers an improved drought tolerance but results in an attenuated cold tolerance in rice, demonstrating that *ONAC095* plays opposite roles in drought and cold stress response.

## Results

### *ONAC095* is a drought- and ABA-up-regulated but cold-down-regulated *NAC* gene

**Fig. 1:**
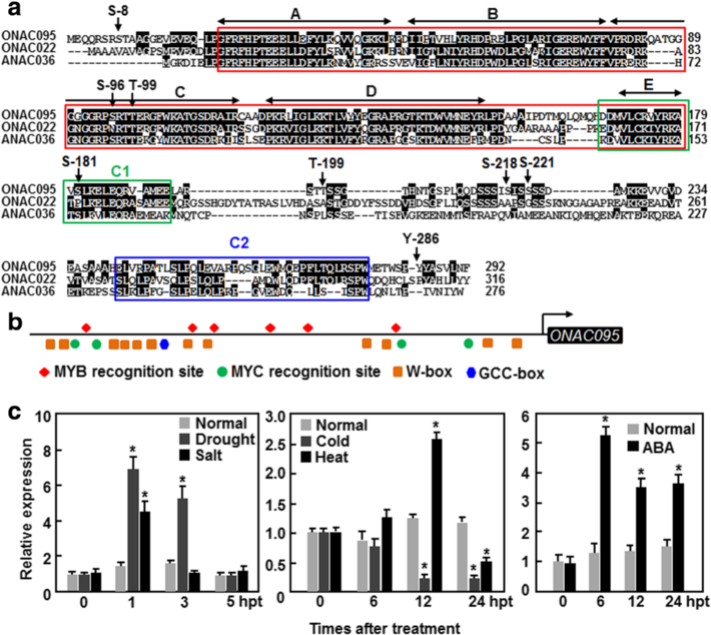
Structural feature of ONAC095, *cis*-elements in promoter of *ONAC095* and stress-responsive expression of *ONAC095*. **a** Alignment of ONAC095 with rice ONAC022 and Arabidopsis ANAC036. The conserved NAC domain is boxed in red and the five highly conserved subdomains A to E are indicated by black arrowed lines. C1 and C2 domains are boxed with green and blue lines, respectively. Putative phosphorylation sites are indicated by arrows. S, serine; T, threonine; Y, tyrosine. **b** Distribution of major stress-related *cis*-elements in the promoter (1.5 Kb upstream of the start codon) of the *ONAC095* gene. **c** Stress-responsive expression of *ONAC095* by drought, salt, cold and heat stresses as well as by exogenous ABA. Drought stress was applied to two-week-old seedlings by transferring the seedlings to three layers filter papers for fast dehydration. Salt stress was applied to the seedlings by rooting the seedlings in 150 mM NaCl solution. Cold and heat stresses were applied by placing the seedlings in growth chambers with temperatures set at 4 °C or 42 °C, respectively. For ABA treatment, the seedlings were sprayed with 100 μM ABA or similar volume of solution as controls. Relative expression levels of *ONAC095* were normalized by the transcript level of the *Actin* gene and the expression level was set as 1 at 0 hr after treatment. Data presented in **c** are the means ± SD from three independent experiments and columns with an asterisk indicate significant difference at *p* < 0.05 level between the treatments and normal controls

The *ONAC095* gene (LOC_Os06g51070) encodes a 292 aa protein with a typical N-terminal NAC domain, which can be divided into 5 subdomains (A to E) [20] (Fig. 1a). Although the sequence outside the NAC domain is divergent, two conserved C1 and C2 domains [43] are present in the C-terminal region of the ONAC095 protein (Fig. 1a). ONAC095 is closely related to rice ONAC022 and Arabidopsis ANAC036, showing 62.4 % and 52.0 % of identity, respectively. The ONAC095 protein contains eight putative phosphorylation sites with probability of >90 %, including five serine (S) residues at positions of 8, 96, 181, 218 and 221 aa, two threonine (T) residues at positions of 99 and 199 aa, and one tyrosine (Y) residue at position of 286 aa (Fig. 1a). Bioinformatics analysis indicated that several stress-responsive *cis*-elements including 1 GCC box, 4 MYC recognition sites, 6 MYB recognition sites and 12 W-boxes are present in the promoter region (1.5 Kb upstream of the start codon) of the *ONAC095* gene (Fig. 1b). We examined by quantitative real time-PCR (qRT-PCR) the responsiveness of *ONAC095* to abiotic stress and ABA. Expression of *ONAC095* in detached leaves was significantly and rapidly up-regulated within 3 hr by fast dehydration, giving 3.9 and 2.2 folds of increase at 1 and 3 hr after drought treatment, respectively (Fig. 1c). An increase of 2.1 folds in *ONAC095* expression level was observed at 1 hr after treatment with 150 mM NaCl but the expression decreased rapidly to basal level at 3 hr after treatment (Fig. 1c). By contrast, the expression of *ONAC095* was down-regulated gradually by cold (4 °c) treatment, showing ~5 folds of decrease at 12 and 24 hr after treatment (Fig. 1c). In heat (42 °c)-treated plants, the expression of *ONAC095* increased within 12 hr and decreased to basal level at 24 hr after treatment (Fig. 1c). Significant induction of *ONAC095* expression in ABA-treated plants was also observed, showing 3.3 folds of increase at 6 hr after treatment (Fig. 1c). These data suggest that *ONAC095* is a drought- and ABA-up-regulated but cold-down-regulated stress-responsive rice *NAC* gene.

### ONAC095 has transactivation activity that is determined by two conserved proline residues in the C-terminal C2 domain

**Fig. 2:**
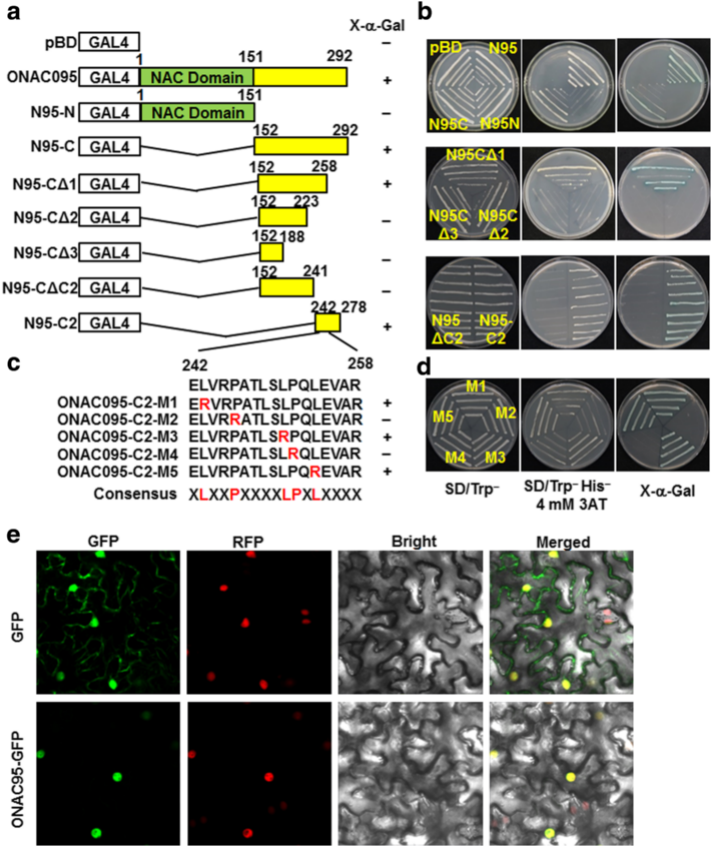
Transactivation activity and nuclear localization of ONAC095. **a**–**d** Transactivation activity and mapping of the specific sequence responsible for transactivation activity in ONAC095. **a** and **c** Diagrams showing different truncated constructs and mutated amino acid residues in C2 domain, respectively. **b** and **d** Transcription activity assay for truncated and mutated constructs of ONAC095 in yeast. Yeasts harboring different truncated and mutated constructs and empty vector were grown on the SD/Trp^−^ plates or SD/Trp^−^ His^−^ with 4 mM 3-AT for 3 days at 30 °C. Transactivation activity was examined by the growth ability and production of blue pigment after addition of X-α-gal in the SD/Trp^−^ His^−^ plates for 1 day. **e** ONAC095 is localized in nucleus. Agrobacteria harboring pFGC-ONAC095 or pFGC-eGFP were infiltrated into leaves of *N. benthamiana* plants expressing a red nucleus marker protein RFP-H2B and leaf samples were collected at 24 hr after agroinfiltration. Microscopic examination was performed under a confocal laser scanning microscope in dark field for green fluorescence (left), red fluorescence (middle left), white field for cell morphology (middle right) and in combination (right), respectively

To examine whether ONAC095 had transactivation activity, the full ONAC095 protein, a C-terminal-truncated N-terminal fragment ONAC095-N (lacking 152–292 aa at C-terminal) and an N-terminal-truncated C-terminal fragment ONAC095-C (lacking 1–151 aa at N-terminal) were each fused to the GAL4 DNA-binding domain in pBD vector (Fig. 2a). Yeasts harboring GAL4-ONAC095, ONAC095-N or ONAC095-C all grew on SD/Trp^−^ medium (Fig. 2b). However, only yeasts harboring GAL4-ONAC095 or GAL4-ONAC095-C grew while yeasts carrying GAL4-ONAC095-N and empty pBD vector did not grow on SD/Trp^−^His^−^ medium containing 4 mM 3-amino-1,2,4-triazole (3-AT) (Fig. 2b). Yeasts harboring GAL4-ONAC095 or GAL4-ONAC095-C showed significant β-galactosidase activity after addition of X-α-gal (Fig. 2b), indicating that ONAC095 had transactivation activity and the C-terminal is responsible for its transactivation activity. We then mapped the putative sequence responsible for transactivation activity in C-terminal by testing a series of truncated C-terminal constructs for their transactivation activity (Fig. 2a). Yeasts carrying GAL4-ONAC095-CΔ1 (lacking 259–292 aa from C-terminal) grew on SD/Trp^−^His^−^ medium and displayed β-galactosidase activity (Fig. 2b). By contrast, yeasts carrying GAL4-ONAC095-CΔ2 (lacking 224–292 aa from C-terminal) or GAL4-ONAC095-CΔ3 (lacking 189–292 aa from C-terminal) did not grow on SD/Trp^−^His^−^ medium and did not show β-galactosidase activity (Fig. 2b), suggesting that the specific sequence between 224–292 aa in C-terminal of ONAC095 is responsible for transactivation activity. To examine the possibility whether a part of the C2 domain may be the determinant responsible for the transactivation activity, we further tested the transactivation activity of truncated constructs GAL4-ONAC095-CΔC2 (lacking 242–292 aa from C-terminal), in which the C2 domain was fully deleted, and GAL4-ONAC095-C2, which spanned 242–278 aa containing the complete C2 domain (Fig. 2a). As shown in Fig. 2b, yeasts harboring GAL4-ONAC095-CΔC2 did not grow on SD/Trp^−^His^−^ medium and did not show β-galactosidase activity while yeasts harboring GAL4-ONAC095-C2 grew on SD/Trp^−^His^−^ medium and showed β-galactosidase activity, confirming that the C2 domain is responsible for transactivation activity of ONAC095. Because yeasts harboring GAL4-ONAC095-CΔ1 had transactivation activity, it is possible that the specific sequence for transactivation activity is located between 242–258 aa of ONAC095, a region containing five conserved amino acid residues in a consensus of xLxxPxxxxLPxLxxxx when aligned with ONAC022 and ANAC036 (Fig. 1a). To determine the importance of these five conserved residues in the transactivation activity, we constructed a series of mutated versions, ONAC095-C2-M1-5, in which the leucine (L) residues at 243, 251 and 254 aa and the proline (P) residues at 246 and 252 aa in 242–258 aa region were individually replaced with arginine (R) (Fig. 2c) and tested for their transactivation activity. As shown in Fig. 2d, yeasts harboring GAL4-ONAC095-C2-M2 or GAL4-ONAC095-C2-M4 did not grow on SD/Trp^−^His^−^ medium and did not show β-galactosidase activity, whereas yeasts harboring GAL4-ONAC095-C2-M1, GAL4-ONAC095-C2-M3 or GAL4-ONAC095-C2-M5 did grow and show β-galactosidase activity, demonstrating that the conserved proline residues at 246 and 252 aa are critical and required for the transactivation activity of ONAC095.

### ONAC095 is a nucleus-localized protein

To examine the subcellular localization of ONAC095, the coding sequence of *ONAC095* was fused in-frame with GFP at N-terminal in pFGC-EGFP vector and transiently expressed in leaves of *Nicotiana benthamiana* plants harboring a red nuclear marker RFP–H2B protein [44]. Microscopic observations of the agroinfiltrated *N. benthamiana* leaves collected at 24 hr after agroinfiltration revealed that the GFP:ONAC095 fusion was solely localized in nucleus, co-localized with the known nuclear marker RFP–H2B protein (Fig. 2e), whereas GFP alone distributed ubiquitously throughout the cell without specific compartmental localization (Fig. 2e). These data indicate that ONAC095 is a nucleus-localized protein.

### Generation and characterization of *ONAC095* overexpression and dominant chimeric repressor-mediated suppression transgenic lines

**Fig. 3:**
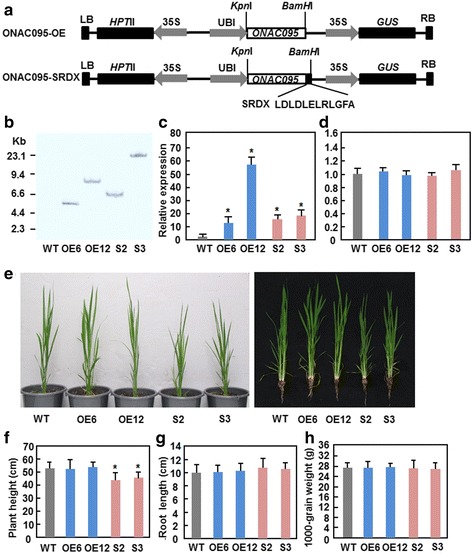
Characterization of ONAC095-OE and ONAC095-SRDX transgenic rice lines and their growth phenotypes. **a** Schematic diagrams showing the overexpression ONAC095-OE and the dominant chimeric repressor-mediated suppression ONAC095-SRDX constructs used for transformation. *Hpt*II, hygromycin phosphotransferase II; LB, left border; RB, right border; Ubi, maize ubiquitin promoter; 35S, CaMV 35S promoter; *GUS*, β-glucuronidase. **b** Confirmation of single-copy transgenic lines by Southern blot analysis. Fifty micrograms of genomic DNA were digested with *Eco*RI and probed with a DIG-labeled fragment of the *Hpt*II gene. **c** Transcript levels of *ONAC095* and *ONAC095-SRDX* in ONAC095-OE and ONAC095-SRDX transgenic lines. Leaf samples from two-week-old seedlings were used for analysis of the transcript levels by qRT-PCR. **d** Transcript levels of *ONAC022* in ONAC095-OE and ONAC095-SRDX transgenic lines. Leaf samples from two-week-old seedlings were used for analysis of the transcript levels by qRT-PCR. **e** Growth phenotype of two-month-old ONAC095-OE and ONAC095-SRDX plants grown under normal watered condition in greenhouse. **f** and **g** Plant height and root length of two-month-old ONAC095-OE and ONAC095-SRDX plants grown under normal watered condition in greenhouse. **h** Weights of 1000-grain from ONAC095-OE and ONAC095-SRDX plants grown under normal watered condition in greenhouse. Data presented (**c**, **d**, **f**, **g**) and (**h**) are the mean ± SD from three independent experiments and columns with an asterisk indicate significant difference at *p* ≤ 0.05 level between WT and OE/SRDX lines. WT, wild type; OE6, ONAC095-OE6; OE12, ONAC095-OE12; S2, ONAC095-SRDX2; S3, ONAC095-SRDX3

To explore the function of *ONAC095* in abiotic stress tolerance, we generated transgenic rice lines with overexpression of *ONAC095* or dominant chimeric repressor-mediated suppression of ONAC095 function. A maize ubiquitin promoter-driven overexpression construct ONAC095-OE was made by inserting the *ONAC095* coding sequence into a modified binary vector PU1301 (Fig. 3a). Considering that functional redundancy often occurs in some of NAC TFs [31, 45], a dominant chimeric repressor-mediated suppression construct ONAC095-SRDX was also made by fusing the *ONAC095* coding sequence at its C-terminal to a plant-specific transcriptional repression domain [46] (Fig. 3a). The ONAC095-OE and ONAC095-SRDX constructs were introduced into rice cv. Xiushui 134 calli through *Agrobacterium*-mediated transformation method. Eighteen independent ONAC095-OE lines and 21 independent ONAC095-SRDX lines were obtained. After screening phenotype and segregation of hygromycin (Hgr) resistance on 1/2 MS medium in T2 and T3 generations, two overexpression lines ONAC095-OE6 and ONAC095-OE12 and two dominant chimeric repressor-mediated suppression lines ONAC095-SRDX2 (S2) and ONAC095-SRDX3 (S3) were identified as single-copy homozygous transgenic lines. Southern blotting of genomic DNA probed with a fragment of the *Hpt*II gene confirmed that each of these selected ONAC095-OE and ONAC095-SRDX lines contained a single copy of the transgenic construct (Fig. 3b). qRT-PCR analysis revealed that the transcript levels of *ONAC095* in T3 generation plants of ONAC095-OE6 and ONAC095-OE12 lines were ~11 and ~57 times higher than that in wild-type (WT) plants, respectively, whereas the transcript levels of *ONAC095-SRDX* in T3 generation plants of ONAC095-SRDX2 and ONAC095-SRDX3 lines were ~14 and ~18 times higher over that in WT, respectively (Fig. 3c). Considering that *ONAC022* is closely related to *ONAC095* [42], we also examined whether altered expression of *ONAC095* in transgenic plants affected the expression of *ONAC022*. qRT-PCR data showed that the expression level of *ONAC022* in ONAC095-OE and ONAC095-SRDX plants was comparable to that in WT (Fig. 3d), indicating that altered expression of *ONAC095* does not affect the expression of *ONAC022* in transgenic rice. We did not observe any difference in plant height and root length between ONAC095-OE and WT plants grown in greenhouse (Fig. 3e–g). However, we noticed that ONAC095-SRDX plants showed growth retardation (Fig. 3e), leading to 11–15 % of reduction in plant height (Fig. 3f), as compared to WT. The root lengths and 1000-grain weights from ONAC095-OE and ONAC095-SRDX plants grown in greenhouse were comparable to WT (Fig. 3g and h). Thus, it is likely that dominant chimeric repressor-mediated suppression of ONAC095 function has a negative impact on rice growth and development.

### Dominant chimeric repressor-mediated suppression of ONAC095 function confers an improved drought tolerance

**Fig. 4:**
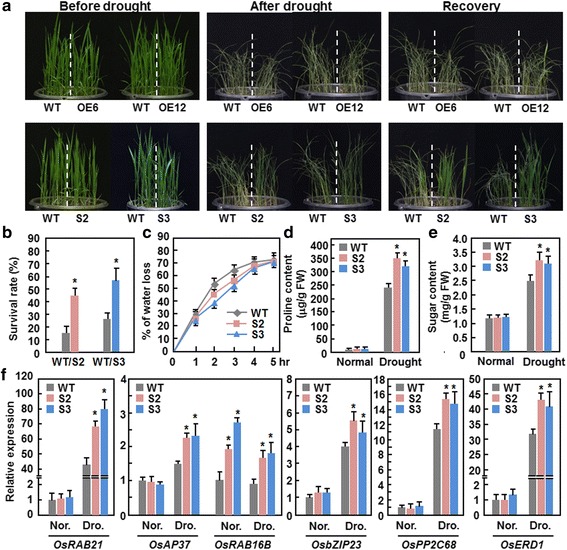
Dominant chimeric repressor-mediated suppression of ONAC095 function conferred an improved drought tolerance in ONAC095-SRDX plants. **a** Growth performance of ONAC095-OE, ONAC095-SRDX and WT plants at different stages during a drought stress experiment. ONAC095-OE and ONAC095-SRDX plants were grown in same barrels with WT and were subjected to drought stress treatment by withholding water for 20 days. Drought stressed plants were recovered for another 7 days by normally watering. **b**–**e** Comparison of survival rate, water loss rate, contents of proline and soluble sugars between ONAC095-SRDX and WT plants with or without drought stress treatment. Plants with >20 % green leaves were considered to be survivals (**b**). Rates of water loss in detached leaves of 4-week-old ONAC095-SRDX and WT plants were measured at indicated time points over a period of 5 hr after detachment (**c**). Leaf samples form 4-week-old ONAC095-SRDX and WT plants grown under normally watered and drought-stressed (at 6 days after water withholding) conditions were collected and measured for contents of proline (**d**) and soluble sugars (**e**). **f** Expression of drought stress-related genes in ONAC095-SRDX and WT plants before and after drought stress treatment. Leaf samples were collected from normally watered and drought stressed plants for 15 days. Relative expression levels were normalized by the transcript level of the *Actin* gene as an internal control and the expression level of each gene of interest in WT plants under normal condition was set as 1. Nor., normally watered condition; Dro., drought stressed condition. Data presented in (**b**–**f**) are the means ± SD from three independent experiments and columns with an asterisk indicate significant difference at *p* < 0.05 level between WT and SRDX lines. WT, wild type; OE6, ONAC095-OE6; OE12, ONAC095-OE12; S2, ONAC095-SRDX2; S3, ONAC095-SRDX3

We first explored the involvement of *ONAC095* in drought tolerance by phenotyping ONAC095-OE and ONAC095-SRDX plants under drought condition and comparing with WT. In our repeated drought stress experiments, drought symptom, represented by rolled leaves and wilted plants, in ONAC095-OE lines at 20 days after drought treatment and at 7 days after recovery of watering was indistinguishable from WT (Fig. 4a), indicating that overexpression of *ONAC095* in transgenic rice does not affect the drought tolerance. By contrast, drought symptom in ONAC095-SRDX plants at 20 days after drought treatment and at 7 days after recovery of watering was markedly less severe than WT (Fig. 4a). At 7 days after recovery of watering, the survival rate of ONAC095-SRDX plants was ~30 % higher than WT (Fig. 4b). To explore the possible mechanism responsible for the improved drought stress tolerance in ONAC095-SRDX plants, we analyzed and compared some stress-related physiological and biochemical changes and the expression of several selected drought stress-responsive genes between ONAC095-SRDX and WT plants grown under normally watered and/or drought stressed conditions. The rate of water loss, as calculated from the relative water content (RWC), in detached leaves of ONAC095-SRDX plants decreased by 9–15 %, as compared with WT, at 2 and 3 hr after detachment (Fig. 4c). Under normally watered condition, the contents of proline and soluble sugars in ONAC095-SRDX plants were comparable to those in WT (Fig. 4d and e). However, the contents of proline and soluble sugars in ONAC095-SRDX and WT plants at 10 days under drought stressed condition were increased significantly as compared to those in plants grown under normally watered condition (Fig. 4d and e). Further, the increase in contents of proline and soluble sugars in ONAC095-SRDX plants was much evident than those in WT under drought stressed condition, resulting in increase of 30–43 % for proline content and 28–31 % for soluble sugar content, respectively (Fig. 4d and e). Similarly, the expression levels of *OsPP2C28*, a member of the PP2C family known to be involved in abiotic stress response [47], *OsbZIP23* and *OsAP37*, two stress-responsive TF genes [48, 49], and *OsRAB21*, *OsRAB16B* and *OsERD1* (a homolog of Arabidopsis *AtERD1*), three stress-related genes [50, 51], in ONAC095-SRDX and WT plants grown under normally watered condition were comparable (Fig. 4f). Under drought stressed condition, the expression of these genes was significantly up-regulated in both ONAC095-SRDX and WT plants compared to those in plants grown under normally watered condition; however, the expression levels in ONAC095-SRDX plants showed a further increase over those in WT (Fig. 4f). Together, these data indicate that dominant chimeric repressor-mediated suppression of ONAC095 function in ONAC095-SRDX plants confers an improved drought stress tolerance that may be resulted from reduced transpiration rate, increased contents of stress-related metabolites, and up-regulated expression of drought-responsive genes.

### Dominant chimeric repressor-mediated suppression of ONAC095 function attenuates cold stress tolerance

**Fig. 5:**
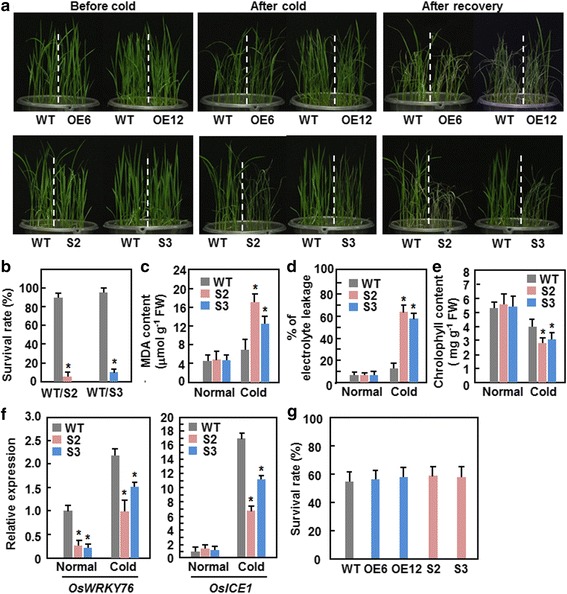
Dominant chimeric repressor-mediated suppression of ONAC095 function in ONAC095-SRDX plants attenuated cold stress tolerance but did not affect heat stress tolerance. **a** Growth performance of ONAC095-OE, ONAC095-SRDX and WT plants at different stages during a cold stress experiment. ONAC095-OE and ONAC095-SRDX plants were grown in same barrels with WT and were subjected to cold stress treatment in a growth chamber with temperature at 4 °C for 5 days for ONAC095-OE/WT plants or for 1 day for ONAC095-SRDX/WT plants. The cold stressed plants were recovered under normal condition for 7 days. **b** Survival rates of the cold stress-treated plants. **c**–**e** Comparison of MDA content (**c**), electrolyte leakage (**d**) and chlorophyll content (**e**) in leaves of ONAC095-SRDX and WT plants with or without cold treatment. **f** Expression of cold stress-related genes in ONAC095-SRDX and WT plants before and after 1 day of cold treatment. Relative expression levels were normalized by the transcript level of the *Actin* gene as an internal control and the expression level of the tested genes in WT plants under normal condition was set as 1. **g** Survival rates of the heat stress-treated plants. Three-week-old WT, ONAC095-OE and ONAC095-SRDX plants were treated with heat stress in a growth chamber with temperature of 45 °C for 5 days and survivals were recorded at 7 days after recovering under normal condition. Data presented in (**b**–**g**) are the means ± SD from three independent experiments and columns with an asterisk indicate significant difference at *p* < 0.05 level between WT and SRDX lines. WT, wild type; OE6, ONAC095-OE6; OE12, ONAC095-OE12; S2, ONAC095-SRDX2; S3, ONAC095-SRDX3

The fact that the expression of *ONAC095* was down-regulated by cold stress led us to examine whether *ONAC095* plays a role in cold stress tolerance by phenotyping ONAC095-OE and ONAC095-SRDX plants under cold stress condition and comparing with WT. In repeated cold stress experiments, ONAC095-OE plants displayed indistinguishable cold stress symptoms such as rolled leaves and wilted plants from those of WT at 5 days after cold (4 °C) treatment and at 7 days after recovery (Fig. 5a), indicating that overexpression of *ONAC095* in transgenic rice does not affect the cold tolerance. By contrast, the ONAC095-SRDX plants showed more severe cold stress symptoms at 7 days after recovery from cold stress than those of WT (Fig. 5a). At 7 days after recovery from cold stress, <10 % of the ONAC095-SRDX plants survived while ~90 % of WT survived (Fig. 5b). To elucidate the possible mechanism responsible for the attenuated cold stress tolerance in the ONAC095-SRDX plants, we analyzed and compared some stress-related physiological changes, e.g. malondialdehyde (MDA) content, electrolyte leakage and chlorophyll content, and the expression changes of several selected cold-responsive genes between ONAC095-SRDX and WT plants grown under unstressed or cold stressed conditions. Under unstressed condition, MDA content, relative electrolyte leakage and chlorophyll content in ONAC095-SRDX plants were comparable to those in WT (Fig. 5c–e). However, MDA content and relative electrolyte leakage in ONAC095-SRDX and WT plants at 1 day under cold stressed condition were increased significantly but chlorophyll content was decreased markedly in ONAC095-SRDX and WT plants as compared to those in plants grown under unstressed condition (Fig. 5c–e). Further, increase in MDA content and relative electrolyte leakage and decrease in chlorophyll content were more evident in ONAC095-SRDX plants than those in WT under cold stressed condition, resulting in increase of 0.9–1.5 folds for MDA content and of 4.3–4.7 folds for relative electrolyte leakage and decrease of 25–30 % for chlorophyll content, respectively (Fig. 5c–e). Under unstressed condition, the expression level of *OsICE1* [52] in ONAC095-SRDX plants was comparable to that in WT but the expression levels of *OsWRKY76* [53] in ONAC095-SRDX plants were significantly lower than that in WT (Fig. 5f). Under cold stressed condition, the expression levels of *OsWRKY76* and *OsICE1* were significantly up-regulated in both ONAC095-SRDX and WT plants compared to those in plants grown under unstressed condition; however, the cold-induced expression levels in ONAC095-SRDX plants were lower than those in WT (Fig. 5f). Collectively, these data indicate that dominant chimeric repressor-mediated suppression of ONAC095 function in ONAC095-SRDX plants attenuates the cold stress tolerance that may be resulted from increased MDA content and down-regulated expression of cold-responsive genes.

We also examined whether altered expression of *ONAC095* affected the thermotolerance in rice by phenotyping ONAC095-OE and ONAC095-SRDX plants under heat stress condition and comparing with WT. In repeated experiments, both the ONAC095-OE and ONAC095-SRDX plants displayed indistinguishable phenotype from WT and similar survival rate to WT after heat stress treatment (Fig. 5g), indicating that overexpression of *ONAC095* or dominant chimeric repressor-mediated suppression of ONAC095 function does not affect the thermotolerance in transgenic rice.

### Dominant chimeric repressor-mediated suppression of ONAC095 function accelerates cold stress-induced ROS accumulation

**Fig. 6:**
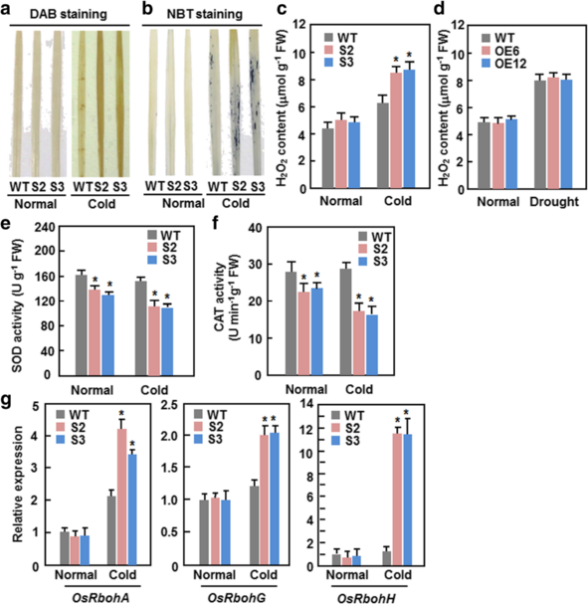
Dominant chimeric repressor-mediated suppression of ONAC095 function accelerated cold-induced ROS accumulation in ONAC095-SRDX plants. Three-week-old ONAC095-SRDX and WT plants were subjected to cold stress treatment by moving into a growth chamber with temperature of 4 °C for 1 day and leaf samples from cold stressed and unstressed plants were collected for different analyses. **a** and **b** In situ detection of H_2_O_2_ and superoxide anion in leaves by DAB and NBT staining, respectively. **c** and **d** Quantification of H_2_O_2_ level in leaves of ONAC095-SRDX and WT plants with or without cold (**c**) or drought (**d**) stress treatment. **e** and **f** Activity of SOD and CAT in leaves of ONAC095-SRDX and WT plants with or without cold stress treatment. **g** Expression of selected *OsRboh* genes in ONAC095-SRDX and WT plants with or without cold stress treatment. Relative expression levels were normalized by the transcript level of the *Actin* gene as an internal control and the expression level of the tested genes in WT plants under normal condition was set as 1. Data presented in (**c**–**g**) are the means ± SD from three independent experiments and columns with an asterisk indicate significant differences at *p* < 0.05 level between WT and OE/SRDX lines. WT, wild type; OE6, ONAC095-OE6; OE12, ONAC095-OE12; S2, ONAC095-SRDX2; S3, ONAC095-SRDX3

Considering that ROS is often linked to oxidative damage during abiotic stress response, we analyzed and compared the generation and accumulation of ROS between ONAC095-SRDX and WT plants grown under unstressed and cold stressed conditions to explore the involvement of ROS in attenuated cold stress tolerance in ONAC095-SRDX plants. In situ detection of ROS by 3,3’-diaminobenzidine (DAB) and nitroblue tetrazolium (NBT) staining and quantitative measurement of H_2_O_2_ revealed that no significant difference in accumulation of H_2_O_2_ and superoxide anion in leaf tissues was observed between ONAC095-SRDX and WT plants grown under unstressed condition (Fig. 6a–d). At 1 days after cold (4 °C) treatment, significant accumulation of H_2_O_2_ and superoxide anion in leaves of both ONAC095-SRDX and WT plants was detected (Fig. 6a–c); however, the accumulation levels of H_2_O_2_ and superoxide anion in leaves of ONAC095-SRDX plants were more evident as compared to those in WT (Fig. 6a and b) or higher than those in WT (Fig. 6c). The levels of H_2_O_2_ in leaves of the ONAC095-SRDX and WT plants increased under drought stress condition; however, the accumulation levels of H_2_O_2_ in leaves of ONAC095-SRDX plants were comparable to that in WT (Fig. 6d). In unstressed plants, the activity of superoxide dismutase (SOD) and catalase (CAT) in ONAC095-SRDX plants was lower than that in WT (Fig. 6e and f); the decrease of SOD and CAT activity in ONAC095-SRDX plants was more evident at 1 day after cold stress treatment, leading to 30 % of decrease in activity, as compared to those in WT (Fig. 6e and f). The expression levels of *OsRbohA*, *OsRbohG* and *OsRbohH* genes, encoding for NADPH oxidases involved in ROS generation [7, 54], were comparable between ONAC095-SRDX and WT plants (Fig. 6g). At 1 day after cold treatment, the expression levels of *OsRbohA*, *OsRbohG* and *OsRbohH* in leaves of ONAC095-SRDX plants increased significantly, showing 0.6–1.0, 0.5–0.6 and 9.2–9.4 folds of increases, respectively, as compared to those in WT (Fig. 6g). Taken together, these data indicate that an abnormal generation and accumulation of ROS, due to up-regulated expression of *OsRboh* genes and reduced activity of antioxidant enzymes, occurs in ONAC095-SRDX plants upon cold stress, and the accumulated ROS may then attenuate the cold stress tolerance via cellular oxidative damage.

### Dominant chimeric repressor-mediated suppression of ONAC095 function increases ABA sensitivity

**Fig. 7:**
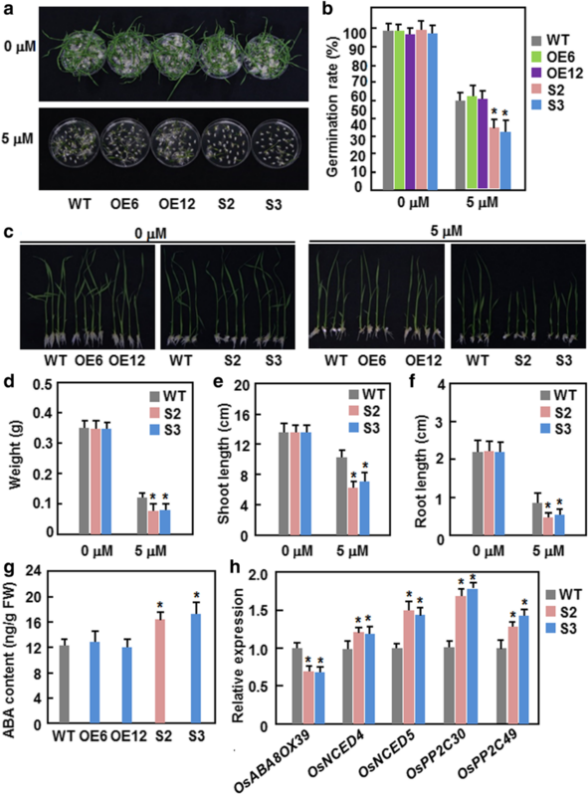
Dominant chimeric repressor-mediated suppression of ONAC095 function increased ABA sensitivity in ONAC095-SRDX lines. **a** and **b** Germination performance and rates of ONAC095-OE, ONAC095-SRDX and WT seeds on 1/2 MS medium supplemented with or without 5 μM ABA. **c**–**f** Growth performance of ONAC095-OE, ONAC095-SRDX and WT seedlings grown on 1/2 MS medium with or without 5 μM ABA. Weight of single seedling (**d**) and length of shoot (**e**) and root (**f**) were measured at 6 days after germination. **g** ABA content in two-week-old ONAC095-OE, ONAC095-SRDX and WT plants grown under normally watered condition. **h** Expression levels of ABA biosynthesis- and metabolism-related and ABA signaling-related genes in ONAC095-SRDX and WT plants. Relative expression levels were normalized by the transcript level of the *Actin* gene as an internal control and the expression level of the tested genes in WT plants under normal condition was set as 1. Data presented in (**b**) and (**d**–**h**) are the means ± SD from three independent experiments and columns with an asterisk indicate significant difference at *p* < 0.05 level between WT and OE/SRDX lines. WT, wild type; OE6, ONAC095-OE6; OE12, ONAC095-OE12; S2, ONAC095-SRDX2; S3, ONAC095-SRDX3

The fact that expression of *ONAC095* was induced by ABA led us to explore whether altered expression of *ONAC095* affected ABA sensitivity in ONAC095-OE and ONAC095-SRDX plants. We first examined the ABA sensitivity of ONAC095-OE and ONAC095-SRDX lines and compared with WT by analyzing seed germination and seedling growth in presence of ABA. In the absence of ABA, seeds of ONAC095-OE, ONAC095-SRDX and WT lines germinated normally and no difference was observed among ONAC095-OE, ONAC095-SRDX and WT (Fig. 7a and b). In the presence of 5 μM ABA, however, germination of ONAC095-OE seeds was comparable to WT but germination of ONAC095-SRDX seeds was significantly inhibited, showing by 25–30 % of decrease, in comparison to WT (Fig. 7a and b). Similarly, growth of ONAC095-OE and ONAC095-SRDX seedlings in the absence of ABA were similar to WT (Fig. 7c); however, in the presence of 5 μM ABA, growth of ONAC095-OE seedlings was comparable to WT but growth of ONAC095-SRDX seedlings was significantly inhibited as compared with WT (Fig. 7c–f). Weight of single seedling, length of shoot and root of ONAC095-SRDX seedlings were decreased by 30–40 % as compared with WT in the presence of 5 μM ABA (Fig. 7d–f). These data indicate that overexpression of *ONAC095* does not affect the ABA sensitivity in ONAC095-OE lines but dominant chimeric repressor-mediated suppression of ONAC095 function in ONAC095-SRDX lines results in increased ABA sensitivity. We next examined whether altered expression of *ONAC095* affected the endogenous ABA level and ABA-mediated signaling in ONAC095-OE and ONAC095-SRDX lines. As shown in Fig. 7g, the endogenous ABA level in ONAC095-SRDX plants was significantly higher than that in WT, leading to 33–42 % of increase; however, no significant difference in ABA content was detected between the ONAC095-OE and WT plants. Accordingly, the expression of ABA biosynthetic gens *OsNCED4* and *OsNCED5* was up-regulated but the expression of an ABA metabolic gene *OsABA8OX39* was down-regulated in ONAC095-SRDX plants grown under normal condition (Fig. 7h). Furthermore, the expression levels of *OsPP2C30* and *OsPP2C49*, two PP2Cs involved in ABA signaling [47], in ONAC095-SRDX plants were also significantly up-regulated as compared to those in WT (Fig. 7h). Together, these results indicate that dominant chimeric repressor-mediated suppression of ONAC095 function affects the endogenous ABA level through regulation of the expression of ABA biosynthetic and metabolic genes and thereby modulates an activated ABA signaling in ONAC095-SRDX plants.

## Discussion

NAC TFs constitute a large family with 151 members in rice [20–22]; however, only a limited number of NAC TFs have been functionally characterized so far. In the present study, we demonstrated through functional analyses using overexpression and dominant chimeric repressor-mediated suppression transgenic lines that *ONAC095* plays opposite roles in drought and cold stress tolerance in rice. Furthermore, biochemical studies revealed that the C2 domain in C-terminal and two proline residues in C2 domain are critical for transactivation activity of ONAC095. Our functional and biochemical studies demonstrate that ONAC095 functions as a dual regulator of abiotic stress response in rice.

NAC TFs consist of a conserved DNA-binding NAC domain, which is responsible for the oligomerization into dimeric proteins [19], and a more divergent C-terminal region, which functions as a transcription regulatory domain [55]. Most of the previously identified NAC TFs were reported to act as transcription activators, although a few of NAC TFs were found to be transcriptional repressors [56, 57]. We showed that ONAC095 is a transcriptional activator, similar to its closely related homologous ONAC022 that can bind specifically to a canonical NAC TF recognition *cis*-element in vitro and act as a transcriptional activator [42]. We also found that the C-terminal of ONAC095 is responsible for its transactivation activity (Fig. 2), consistent with a common concept that NAC TFs have a C-terminal transcriptional activation domain [32, 35, 41, 42]. The ONAC095 protein, similar to its closely related homologous rice ONAC022 [42] and Arabidopsis ANAC036 [43], contains two unique conserved C1 and C2 domains (Fig. 1a), which are not present in other NAC TFs [43]. The C1 domain, consisting of the putative NAC subdomain E and its immediately downstream sequence, was proposed to be involved in DNA-binding ability [43]. In our study, however, the truncated constructs ONAC095-CΔ2 (spanning 152–223 aa) and ONAC095-CΔC2 (spanning 152–241 aa), which harbor the full C1 domain, abolished the transactivation activity (Fig. 2a and b), indicating that the C1 domain is required for transactivation activity. Our biochemical analysis of transactivation activity with a series of truncated constructs of the C-terminal of ONAC095 further identified the possible sequence region and/or critical residues that are responsible for transactivation activity in ONAC095. The truncated construct ONAC095-CΔ1 (spanning 152–258 aa), containing a part of the C2 domain, had transactivation activity but the truncated construct ONAC095-CΔC2 (spanning 152–241 aa), in which the full C2 domain was deleted, did not (Fig. 2a and b), implying that a determinant for transactivation activity exists between 242–258 aa in C-terminal. This is further supported by the observation that the C2 domain itself had transactivation activity (Fig. 2a and b). It was also found that the transactivation activity of SNAC1, which does not have a C2 domain, is located between 242–271 aa [32]. Further mutation analysis revealed that the conserved proline residues at 246 and 252 aa are critical and required for transactivation activity of ONAC095 (Fig. 2c and d). This is consistent with the observation that a single amino acid residue at 270 aa determines the transactivation activity in SNAC1 [32].

Although expression of *ONAC095* was induced by drought stress as well as by ABA (Fig. 1c), dominant chimeric repressor-mediated suppression of ONAC095 function in ONAC095-SRDX plants led to higher survival ratio and better growth performance under drought stress condition (Fig. 4), demonstrating that ONAC095 acts as a negative regulator of drought tolerance in rice. This differs from previously reported rice NAC TFs that play positive roles in improving drought stress tolerance [32–42]. The mechanisms responsible for the improved drought tolerance in ONAC095-SRDX plants can be explained by several physiological, biochemical and molecular changes. Firstly, accumulation of compatible solutes such as soluble sugars and free proline is a common phenomenon in response to abiotic stress [58]. Higher levels of free proline and soluble sugars under drought stress condition (Fig. 4d and e) may partially account for the improved drought and salt tolerance. Secondly, ABA plays a critical role in regulating abiotic stress response in plants [4, 5]. Generally, high level of endogenous ABA may strengthen and/or accelerate stress response and thus correlate with improved abiotic stress tolerance [59, 60]. The ONAC095-SRDX plants contained an elevated level of endogenous ABA (Fig. 7g), which may be resulted from up-regulated expression of ABA biosynthetic genes such as *OsNCEDs* [61] and down-regulated expression of ABA metabolic gene like *OsABA8OX39* [60] (Fig. 7h), and exhibited an increased ABA sensitivity (Fig. 7a–f). Thirdly, an increased endogenous ABA level in plants generally initiates ABA-mediated signaling pathway that regulates the expression of many stress-responsive genes [62]. The expression of some early ABA signaling and regulatory genes such as *OsPP2C30*/*49*/*68* [47], *OsbZIP23* [48] and *OsAP37* [49] and late ABA-responsive genes such as *OsRAB16B* and *OsRAB21* [50], was significantly up-regulated in ONAC095-SRDX plants (Figs. 4f and 7h), implying an activated ABA-mediated signaling pathway in the transgenic plants. These data suggest that dominant chimeric repressor-mediated suppression of ONAC095 function in ONAC095-SRDX plants may modify an ABA-mediated signaling pathway to regulate the expression of stress-responsive genes and thereby confer an improved drought tolerance.

Expression of *ONAC095* was down-regulated by cold stress (Fig. 1c) and the ONAC095-SRDX plants showed severely wilted appearance, lower survival rate and higher percentage of electrolyte leakage after cold treatment (Fig. 5), indicating that dominant chimeric repressor-mediated suppression of ONAC095 function led to an attenuated cold tolerance. The ONAC095-SRDX plants accumulated more H_2_O_2_ and superoxide anion under cold stress (Fig. 6a–c), implying that dominant chimeric repressor-mediated suppression of ONAC095 function may lose the ability to control the balance between the cold-induced ROS generation and scavenging ability. This is supported by the observations that expression of 3 *OsRboh* genes, coding for NADPH oxidases involved in ROS generation [7, 54], were up-regulated while the activities of SOD and CAT, two ROS scavenging enzymes, were decreased in ONAC095-SRDX plants (Fig. 6e–g). Excessive accumulation of ROS often causes the production of MDA, which is toxic to plant cells at high level [8]. After cold stress, ONAC095-SRDX plants had significantly higher MDA level (Fig. 5c). Thus, it is reasonable that abnormal ROS accumulation resulted from imbalanced generation and scavenging may be one of the mechanisms that cause attenuated clod tolerance in ONAC095-SRDX plants. On the other hand, it is generally accepted that plant response to cold stress, like that to drought stress, often requires both ABA-dependent and ABA-independent signaling pathways [63]. The CBF-mediated signaling pathway is believed to be an ABA-independent signaling pathway [64]. ONAC095-SRDX plants contained an elevated ABA level (Fig. 7g) and showed down-regulated expression of *OsICE1* (Fig. 5f), an upstream TF that regulates the expression of *CBF* genes [52]. These results indicate that an ABA-independent rather than ABA-dependent signaling is involved in ONAC095-regulated cold response in ONAC095-SRDX plants, probably partially through the CBF signaling pathway.

Given that plants are frequently exposed to a multitude of environmental stresses complicated molecular mechanisms, both coordinately and separately, that ensure general adaptive responses are essential for plants to survive under stress conditions. It has been shown that cross-talks at different levels between cold and drought signaling pathways occur in plant cells upon perception of environmental cues [4]. Our results in the present study show that dominant chimeric repressor-mediated suppression of ONAC095 function improves drought tolerance but attenuates cold tolerance in transgenic rice plants (Figs. 4 and 5), demonstrating that *ONAC095* acts as a negative regulator of drought response but functions as a positive regulator of cold response. Such opposite roles in cold and drought response indicate that *ONAC095* may be involved in the negative cross-talk between cold and drought responses. Similar observations were also reported for rice *OsGH3-2* and Arabidopsis *AtATL78*, which play opposite roles in drought and cold stress tolerance [65, 66]. On the other hand, it is well known that plants operate specific signaling pathways in response to cold and drought stresses, which differentially activate distinct subsets of stress-related genes [9, 64]. In this regard, it is likely that ONAC095, as a transcriptional activator, may regulate different target genes upon perception of drought and cold stress signals and thereby exert its differential functions in drought and cold stress response. Further comparative analysis of gene expression profiling between ONAC095-SRDX and WT plants under drought and cold stress conditions will provide insights into the *ONAC095*-regulated signaling pathways.

ONAC095 is closely related to rice ONAC022 and Arabidopsis ANAC036 [42]; however, the function and action mode of ONAC095 differ from ONAC022 and ANAC036. Overexpression of *ONAC022* or *ANAC036* in transgenic plants resulted in a dwarf phenotype [42, 43], while dominant chimeric repressor-mediated suppression of ONAC095 function led to a stunted growth phenotype (Fig. 3e and f), indicating that *ONAC095* is required for normal growth and development in rice. On the other hand, the *ONAC022*-overexpressing rice plants contained an elevated ABA level and conferred an improved drought and salt tolerance [42] while the *ONAC095*-suppressed rice plants showed an increased drought stress tolerance and ABA level (Figs. 4a, b and Fig. 7g). Thus, it is likely that ONAC095 and ONAC022, two closely related NAC TFs, have distinguishable biological functions in abiotic stress response through distinct mechanisms.

*ONAC095* was induced by dehydration but repressed by cold stress (Fig. 1c). It is reasonable to speculate that the ONAC095-OE plants with increased expression level of *ONAC095* (Fig. 3c) would mimic “tolerance” to drought stress and should “increase” drought stress tolerance, while they would be more “sensitivity” to cold stress and should “decrease” cold stress tolerance. In contrast, the ONAC095-SRDX plants, in which the ONAC095 function was suppressed by a dominant chimeric repressor, would mimic “sensitivity” to drought stress and should “decrease” drought stress tolerance, while they would also mimic “tolerance” to cold stress and should “increase” cold stress tolerance. Our experimental data indicate that, whereas the ONAC095-SRDX plants did exhibit clear phenotype under drought and cold stress, the ONAC095-OE plants did not show any altered response to drought and cold stress (Figs. 4a and 5a). One possibility is that ONAC095 is necessary but not sufficient to its function in growth/development and abiotic stress response. Alternatively, post-translational modification such as phosphorylation may be required for the function of the ONAC095 protein. This is partially supported by the presence of several putative phosphorylation sites with probability of >90 % in ONAC095 protein (Fig. 1a). If this is the case, it is then reasonable that simply overexpression of the *ONAC095* gene in transgenic rice should not be enough to confer an altered phenotype. Recently, it was reported that transgenic rice plants overexpressing a phosphomimicking mutated OsWRKY53 showed further-enhanced disease resistance than the native OsWRKY53-overexpressing rice plants [67]. Thus, detailed biochemical assays are required to examine whether ONAC095 can be phosphorylated, and if so, to determine the putative phosphorylation sites. Once the phosphorylation feature is established, creating transgenic lines with overexpression of phosphomimicking mutated version of ONAC095 and examining the phenotype under abiotic stress including drought stress will clarify whether post-translational phosphorylation is required for the function of ONAC095. Additionally, it is also possible that the choice of the constitutive ubiquitin promoter to control the expression of *ONAC095* in overexpression transgenic lines led to malfunction of ONAC095, thereby resulting in indistinguishable phenotype in ONAC095-OE plants from WT plants under drought and cold stress. Surprisingly, neither the ONAC095-OE nor the ONAC095-SRDX plants exhibited altered response to heat stress (Fig. 5g), although expression of *ONAC095* was significantly induced by heat stress (Fig. 1c).

Expression of *ONAC095* was affected differentially in response to drought, salt, cold and heat stress (Fig. 1c); however, no typical abiotic stress-related *cis*-element like DREB or ABRE was identified in the *ONAC095* promoter (Fig. 1b). One possibility is that the promoter of *ONAC095* may contain unidentified novel abiotic stress- and/or ABA-related *cis*-elements that drive its expression in response to abiotic stress. Similar observations were reported for some stress-responsive rice *ONAC* genes. For example, although there are no predicted stress-related *cis*-elements in promoters of the *OsNAC4*, *OsNAC5* and *OsNAC6* genes, all these three *ONAC* genes respond to abiotic stress [35]. Alternatively, the responsiveness of *ONAC095* to abiotic stress is modulated by other TFs via the stress-related *cis*-elements like W-box and GCC-box in the promotor region of *ONAC095* (Fig. 1b). In this regard, *ONAC095* may function during relatively late stage in the stress response network [10]. Further detailed analysis of the *ONAC095* promoter and its *cis*-elements will provide new insights into the regulation mechanism of *ONAC095* expression during stress response.

Because ONAC095 is a transcriptional activator rather than a transcriptional repressor, it is unlikely that *ONAC095* directly activates the expression of the ABA- and drought-related genes in ONAC095-SRDX plants; instead, it may suppress the expression of negative regulators for these genes during drought stress. By contrast, *ONAC095* may directly regulate its target genes to modulate basal cold stress tolerance as dominant chimeric repressor-mediated suppression of ONAC095 function in ONAC095-SRDX plants led to down-regulation of some cold-responsive genes. On these regards, it is likely that ONAC095 may regulate different sets of genes that act separately in drought and cold tolerance.

## Conclusion

ONAC095 is a transcriptional activator and the C2 domain in C-terminal and two proline residues in C2 domain are critical for transactivation activity of ONAC095. Functional analyses of the dominant chimeric repressor-mediated suppression transgenic lines demonstrate that ONAC095 acts as a negative regulator of drought response but as a positive regulator of cold response in rice. Further RNA-seq analysis of the ONAC095 regulon and chromatin immunoprecipitation-based identification of downstream target genes will provide new insights into how *ONAC095* differentially regulates the drought and cold tolerance in rice. Although ONAC095 plays opposite roles in drought and cold stress tolerance, our knowledge that dominant chimeric repressor-mediated suppression of ONAC095 function improves drought tolerance can be used to generate drought-tolerant rice germplasms or materials for potentially application in temperate regions.

## Methods

### Plant materials, growth conditions and treatments

Rice (*Oryza sativa* L.) cv. Yuanfengzao (provided by Professor Rongyao Chai, Zhejiang Academy of Agricultural Sciences, Hangzhou, China) was used for analyses of gene expression in response to abiotic stress and ABA treatment while cv. Xiushui 134 (provided by Professor Rongyao Chai, Zhejiang Academy of Agricultural Sciences, Hangzhou, China), which was established for genetic transformation with high frequency in our lab [42], for generation of transgenic lines and phenotype analyses. Seeds were pre-germinated in water for 2 days and the germinated seeds were then planted into a soil mix. All rice plants were grown in a growth room with a cycle of 26 °C 14 hr light (>3000 lux)/22 °C 10 hr dark or in a greenhouse with natural sunlight. For ABA treatment, two-week-old seedlings were sprayed with 100 μM ABA in 0.1 % ethanol solution or with 0.1 % ethanol solution as controls. Drought treatment was applied by placing two-week-old seedlings into three layers of filter papers for fast dehydration and salt treatment was given by rooting the seedlings in a 150 mM NaCl solution. For extreme temperature stress treatments, seedlings were transferred to a growth chamber with temperature at 4 °C for cold treatment or a growth chamber with temperature at 42 °C for heat treatment. Samples were collected at different time points after treatment and stored at −80 °C until use.

### Cloning and bioinformatics analysis of *ONAC095*

Coding sequence of *ONAC095* was amplified with primers of ONAC095-F and ONAC095-R (Additional file 1: Table S1) designed based on the predicted cDNA in Rice Genome Annotation database and cloned into pMD19-T vector, yielding plasmid pMD19-ONAC095. Multiple sequence alignment was performed using ClustalW program in the LaserGene software [68]. The promoter sequence (1500 bp upstream from the transcription start site) of the *ONAC095* gene was searched for putative *cis*-elements at the PlantCARE database (http://bioinformatics.psb.ugent.be/webtools/plantcare/html/) [69]. Putative phosphorylation sites were searched at the NetPhos 2.0 Server (http://www.cbs.dtu.dk/services/NetPhos/) [70].

### Transactivation activity and subcellular localization assays

For analysis of the transactivation activity, the coding sequence and the truncated and mutated sequences of *ONAC095* were obtained by PCR with different pairs of gene-specific primers (Additional file 1: Table S1) and cloned into pBD at *Eco*RI/*Bam*HI sites [71]. The recombinant plasmids and pBD empty vector were transformed into yeast strain AH109. The transformed yeasts were plated on SD/Trp^−^ medium or SD/Trp^−^ His^−^ medium containing 4 mM 3-AT and incubated for 3 days at 30 °C. Transactivation activity was assessed according to the growth status and production of blue pigment after addition of X-α-gal (5-bromo-4-chloro-3-indolyl-α-D-galactopyranoside) on SD/Trp^−^ His^−^ medium. For analysis of the subcellular localization, the coding sequence of *ONAC095* was amplified using primers of ONAC095GFP-F and ONAC095GFP-R (Additional file 1: Table S1) and cloned into pFGC-EGFP at *Bam*HI/*Xba*I sites [72], yielding plasmid pFGC-GFP-ONAC095. Agrobacteria harboring pFGC-GFP-ONAC095 or pFGC-EGFP were infiltrated separately into leaves of *N. benthamiana* plants expressing a nuclear marker RFP–H2B protein [44] (provided by Dr. Michael Goodin, Department of Plant Pathology, University of Kentucky, USA). The agroinfiltrated leaves were collected at 2 days after agroinfiltration and GFP fluorescence signals were detected under a Zeiss LSM 510 Meta confocal laser scanning microscope (Oberkochen, Germany) using a 500–530 nm emission filter [73].

### Binary vector construction, rice transformation and characterization of the transgenic lines

To construct the overexpression vector, the coding sequence of *ONAC095* was amplified with primers of ONAC095OE-F and ONAC095OE-R (Additional file 1: Table S1) and cloned into a modified pCAMBIA1301 vector PU1301 under the control of the maize ubiquitin promoter [74], yielding PU1301-ONAC095-OE. To construct the chimeric suppression vector, the *ONAC095* coding sequence without the stop codon was amplified using the forward primer ONAC095OE-F and the reverse primer ONAC095SRDX-R, which contains a synthetic SRDX (LDLDLELRLGFA) coding sequence fused at the C-terminus [46], and cloned into PU1301, yielding PU1301-ONAC095-SRDX. The resulting constructs PU1301-ONAC095-OE and PU1301-ONAC095-SRDX were introduced into calli of rice cv. Xiushui134 through standard *Agrobacterium*-mediated transformation protocol [75]. Putative single-copy ONAC095-OE and ONAC095-SRDX transgenic lines were screened according to a 3:1 segregation of Hgr^R^ : Hgr^S^ by planting seeds of T2 generation on 1/2 MS medium containing 50 μg/L Hgr. Homozygous single-copy ONAC095-OE and ONAC095-SRDX transgenic lines were selected based on phenotype of 100 % Hgr^R^ for seeds of T3 generation on 1/2 MS medium containing 50 μg/L Hgr. To confirm these single-copy transgenic lines, genomic DNA was extracted using the CTAB procedure [76] and 50 μg of genomic DNA was digested with *Eco*RI. After separation by electrophoresis on a 0.8 % agarose gel, DNAs in gel were transferred by capillary action onto a Hybond-N^+^ nylon membrane (Amersham Biosciences, Little Chalfont, UK) and hybridized with a 589 bp *Hpt*II probe labelled with DIG by the random priming method using a DIG High Prime DNA Labeling and Detection kit (Roche Diagnostics, Shanghai, China). Detection of DIG signals was performed according to the manufacturer’s recommendation.

### Phenotype analyses for abiotic stress tolerance and ABA sensitivity

For drought stress assay, three-week-old ONAC095-OE and ONAC095-SRDX plants were grown with WT plants in same barrels and were subjected to drought stress by withholding water for 20 days, followed by recovery with normal water supply for another 7 days [42]. For cold stress assay, three-week-old ONAC095-OE and ONAC095-SRDX plants were grown with WT plants in same barrels and then transferred into a growth chamber with temperature at 4 °C with a cycle of 16 hr light/8 hr dark for 5 days for ONAC095-OE/WT plants and for 1 day for ONAC095-SRDX/WT plants, followed by transferring to the growth room with normal condition for recovery. For heat tolerance assay, three-week-old ONAC095-OE and ONAC095-SRDX plants were grown with WT plants in same barrels and were transferred into a growth chamber with temperature at 45 °C with a cycle of 16 hr light /8 hr dark for 5 days. After heat treatment, the plants were recovered at 28 °C for 7 days [77]. Plants with >20 % green leaves were considered to be survivals, and the others were considered to be dead plants. Survival rates were calculated as the percentage of survivals in the total plants used in the experiments. In abiotic stress assays, eight plants for each of the transgenic and WT lines were included in a single replicate and four replicates were set for each of the experiments. For ABA sensitivity assay, 60 seeds were plated on 1/2 MS medium with or without 5 μM ABA under 28 °C/25 °C (day/night) with a 12 hr photoperiod. Seed germination was recorded at 6 days after plating and weight of single seedling and length of shoot and root were measured at 10 days after germination [42].

### Physiological and biochemical measurements

Samples for physiological and biochemical measurements except the RWC assay were collected from the drought and cold stress assays. RWC in detached leaves was measured according to a previously reported method [78]. Briefly, fully expanded leaves of three-week-old ONAC095-SRDX and WT plants were detached to record the leaf fresh weight (W_F_), turgid leaf weight (W_T_) and dry weights (W_D_), and RWC was calculated from the equation RWC (%) = (W_F_ − W_D_)/(W_T_ − W_D_) × 100 %. Electrolyte leakage was measured following a modified method [38]. Measurement of chlorophyll content was performed as described previously [79] using 0.5 g of leaf samples and the chlorophyll content was calculated according to the formula Chl (A + B) = 5.24 × A664 + 22.24 × A648. Quantification of MDA content was performed following a previously described protocol [38] using 0.2 g leaf samples. Free proline was determined using colorimetric method [80] with 0.5 g leaf sample and total soluble sugars was measured as previously described [81] using anthrone reagent with 0.5 g leaf sample. Measurement of H_2_O_2_ was followed by a previously described protocol [82] using trichloroacetic acid reagent with 0.5 g leaf sample. Quantification of ABA was performed by a HPLC-Triple quadrupole liquid chromatography-mass spectrometry system (Model 1290/6460, Aglient Technologies, Santa Clara, CA) according to a previously described method [83]. Activity of SOD and CAT was determined spectrophotometrically according to previously described methods [84]. In situ detection of H_2_O_2_ and superoxide anion in leaf tissues was performed by DAB staining [85] and NBT staining [86], respectively.

### qRT-PCR analysis of gene expression

Total RNA was extracted using TRIzol reagent (Invitrogen, Shanghai, China) according to the manufacturer’s instruction. First-strand cDNA was synthesized from 1 μg of total RNA with SuperScript III Kit (Invitrogen, Shanghai, China) according to the manufacturer’s instruction. qRT-PCR reaction contained 12.5 μL SYBR premix Ex TaqTM (TaKaRa, Dalian, China), 1 μL cDNA sample and 10 μM each primer in a final volume of 25 μL and was performed on a CFX96 Real-time System (Bio-Rad, Hercules, CA, USA). A rice Actin gene (accession number KC140129) was used as an internal control to normalize the data and relative expression levels of genes of interest were calculated using the 2^-ΔΔCT^ method. Gene-specific primers used in qRT-PCR are listed in Additional file 1: Table S1.

### Statistical analysis

All experiments were repeated independently for at least three times and data are shown as mean ± SD of three independent experiments. Data were subjected to statistical analysis according to the Student’s *t*-test and the probability values of *p* < 0.05 were considered as significant difference.

## Additional file

Additional file 1:
**Table S1.** Primers used in this study for different purposes. (DOC 76 kb)

## Abbreviations

ABA: Abscisic acid
CAT: Catalase
DAB: 3, 3’-diaminobenzidine
MDA: Malondialdehyde
NBT: Nitroblue tetrazolium
qRT-PCR: Quantitative reverse transcription PCR
ROS: Reactive oxygen species
SOD: Superoxide dismutase
TF: Transcription factor
