# Supplementary material for: Rice NAC transcription factor ONAC095 plays opposite roles in drought and cold stress tolerance
Source: BMC Plant Biol. 2016 Sep 20;16:203. doi: 10.1186/s12870-016-0897-y (PMC5029094; doi:10.1186/s12870-016-0897-y)
Supplement: Additional file 1: — Table S1. Primers used in this study for different purposes. (DOC 76 kb) [file 12870_2016_897_MOESM1_ESM.doc]

**Additional file: Table S1 Primers used in this study for different purposes**

| Primers | Sequences(5′-3′) |
| --- | --- |
| **Cloning** | |
| ONAC095-F | ATGGAGCAGCAGCGGAGCAG |
| ONAC095-R | TCAGAAGTTGAGGACGCTGG |
| **Rice transformation** | |
| ONAC095OE-F | CGGGGTACC ATGGAGCAGCAGCGGAGCAG |
| ONAC095OE-R | CGCGGATCC TCAGAAGTTGAGGACGCTGG |
| ONAC095SRDX-R | CGCGGATCCTTAGGCAAATCCAAGGCGGAGCTCCAG ATCTAGGTCGAG GAAGTTGAGGACGCTGG |
| **Subcellular localization** | |
| ONAC095GFP-F | CGCGGATCC ATGGAGCAGCAGCGGAGCAG |
| ONAC095GFP-R | TGCTCTAGA TCAGAAGTTGAGGACGCTGG |
| **Transactivation activity** | |
| ONAC095pBD-F | AGTGAATTC ATGGAGCAGCAGCGGAGCAG |
| ONAC095pBD-R | AGTGTCGAC TCAGAAGTTGAGGACGCTGG |
| ONAC095pAD-F | AGTGAATTC ATGGAGCAGCAGCGGAGCAG |
| ONAC095pAD-R | AGTGTCGAC TCAGAAGTTGAGGACGCTGG |
| ONAC095NpBD -R | AGTGTCGAC GAGGCGGTACTCGTTCATGA |
| ONAC095CpBD -F | CCGGAATTC CCCGACGCCGCCGCCATT |
| ONAC095CΔ1pBD -R | AGTGTCGAC CCTCGCCACCTCCAGCTGCG |
| ONAC095CΔ2pBD -R | AGTGTCGAC CGAGGAGGAGGAGATGGAGA |
| ONAC095CΔ3pBD -R | AGTGTCGAC CCTCTGCTCCAGCTCCTTGA |
| ONAC095ΔC2pBD -R | CGCGGATCC ATGCGCCGCCGCCGACGCCT |
| ONAC095C2pBD -F | CCGGAATTC GAGCTGGTCCGCCCGGCGAC |
| ONAC095C2pBD -R | CGCGGATCC CCATGGGCTCCGCAGCTGCG |
| ONAC095ΔC2M1pBD -F | CCGGAATTC GAGCGGGTCCGCCCGGCGACGC |
| ONAC095ΔC2M2pBD -F | CCGGAATTC GAGCTGGTCCGCCGGGCGACGC |
| ONAC095ΔC2M3pBD -F | CCGGAATTCGAGCTGGTCCGCCCGGCGACGCTGAGCCGGCCGCAG |
| ONAC095ΔC2M4pBD -F | CCGGAATTCGAGCTGGTCCGCCCGGCGACGCTGAGCCTGCGGCAGCT |
| ONAC095ΔC2M5pBD -F | CCGGAATTCGAGCTGGTCCGCCCGGCGACGCTGAGCCTGCCGCAGCGGGAGGT |
| **qRT-PCR** | |
| ONAC095-RT-F | CATGAAGAAAGAGGTGGTGGGG |
| ONAC095-RT-R | GTCAGGAATGGCTCCTGCATC |
| ONAC022-RT-F | AAGGAGGACATGGTGCTCTGCAAG |
| ONAC022-RT-R | TGCATCTCTTCCATTGCCGAGGCT |
| OsbZIP23-RT-F | GGAGCAGCAAAAGAATGAGG |
| OsbZIP23-RT-R | GGTCTTCAGCTTCACCATCC |
| OsRAB21-RT-F | CCACGGCACCGGGATGACC |
| OsRAB21-RT-R | AGCTTCTCCTTGATCTTGTCCA |
| OsAP37-RT-F | AAGTGACTCCGACTCCTCGTC |
| OsAP37-RT-R | GTTCAGATCCAGATCGAAAGCT |
| OsRAB16B-RT-F | CGGGTAAACAATAAAGTCGTGATG |
| OsRAB16B-RT-R | GCGCACTTACATACAGTGCTACGT |
| OsPP2C68-RT-F | CGCAGCTCCGACAACATCT |
| OsPP2C68-RT-R | GCTGGGTGACACTCTCTCTACAAG |
| OsERD1-RT-F | ACTGTAGTATTACTTGATGAGATA |
| OsERD1-RT-R | CAATATTTGATGTCATGACAAT |
| OsICE-RT-F | GTACTGTACTACCAGTACAGCAG |
| OsICE-RT-F | CCATTAATTGCTTGAAGTCACAA |
| OsWRKY76-RT-F | ACCTCAAGGAGGTGTGCCG |
| OsWRKY76-RT-R | GCAGCTTCTGGAGGATCGC |
| OsRbohA-RT-F | GAGTGGAGGGCATCACTGGGG |
| OsRbohA-RT-R | AACCAAGCTACGGCGGAACCA |
| OsRbohG-RT-F | TGGGTTACCCGGGAGCCATT |
| OsRbohG-RT-R | AGCAGAGTTGTCCGCGCATC |
| OsRbohH-RT-F | CCATTCGAGTGGCACCCGTT |
| OsRbohH-RT-R | AGCTCGAGGTCCAGTCACCG |
| OsABA8OX3-RT-F | ATGGCCTTCTCGCGAAATT |
| OsABA8OX3-RT-R | ATCACCGTTCTGGCAACCA |
| OsNCED4-RT-F | GATTGCACGGCACCTTCATT |
| OsNCED4-RT-R | CTCTGTAATTTGATTTTTCACTGGCTAAT |
| OsNCED5-RT-F | CCCAGCTTGAAGCTTTTGCT |
| OsNCED5-RT-R | ACAACACTGCAACTATCCCTATCACT |
| OsPP2C30-RT-F | CCATCAGACATACTACTC |
| OsPP2C30-RT-R | GATCACATAATTCGGAAC |
| OsPP2C49-RT-F | GGCTTATTCTCTTCCTCCTCTAT |
| OsPP2C49-RT-R | AGTAAATTCTTTGCGACGATGAT |
| OsACTIN-RT-F | ATCCTTGTATGCTAGCGGTCGA |
| OsACTIN-RT-F | ATCCAACCGGAGGATAGCATG |
